# Effects of betaine on lipopolysaccharide-induced memory impairment in mice and the involvement of GABA transporter 2

**DOI:** 10.1186/1742-2094-8-153

**Published:** 2011-11-04

**Authors:** Masaya Miwa, Mizuki Tsuboi, Yumiko Noguchi, Aoi Enokishima, Toshitaka Nabeshima, Masayuki Hiramatsu

**Affiliations:** 1 Laboratory of Neuropsychopharmacology, Graduate School of Environmental and Human Sciences, Meijo University, 150 Yagotoyama, Tenpaku-ku, Nagoya 468-8503, Japan; 2 Department of Chemical Pharmacology, Faculty of Pharmaceutical Sciences, Meijo University, 150 Yagotoyama, Tenpaku-ku, Nagoya 468-8503, Japan

## Abstract

**Background:**

Betaine (glycine betaine or trimethylglycine) plays important roles as an osmolyte and a methyl donor in animals. While betaine is reported to suppress expression of proinflammatory molecules and reduce oxidative stress in aged rat kidney, the effects of betaine on the central nervous system are not well known. In this study, we investigated the effects of betaine on lipopolysaccharide (LPS)-induced memory impairment and on mRNA expression levels of proinflammatory molecules, glial markers, and GABA transporter 2 (GAT2), a betaine/GABA transporter.

**Methods:**

Mice were continuously treated with betaine for 13 days starting 1 day before they were injected with LPS, or received subacute or acute administration of betaine shortly before or after LPS injection. Then, their memory function was evaluated using Y-maze and novel object recognition tests 7 and 10-12 days after LPS injection (30 μg/mouse, i.c.v.), respectively. In addition, mRNA expression levels in hippocampus were measured by real-time RT-PCR at different time points.

**Results:**

Repeated administration of betaine (0.163 mmol/kg, s.c.) prevented LPS-induced memory impairment. GAT2 mRNA levels were significantly increased in hippocampus 24 hr after LPS injection, and administration of betaine blocked this increase. However, betaine did not affect LPS-induced increases in levels of mRNA related to inflammatory responses. Both subacute administration (1 hr before, and 1 and 24 hr after LPS injection) and acute administration (1 hr after LPS injection) of betaine also prevented LPS-induced memory impairment in the Y-maze test.

**Conclusions:**

These data suggest that betaine has protective effects against LPS-induced memory impairment and that prevention of LPS-induced changes in GAT2 mRNA expression is crucial to this ameliorating effect.

## Background

Betaine (glycine betaine or trimethylglycine) is widely distributed in plants and microorganisms as well as in various dietary sources [1,2]. Some plants accumulate high levels of betaine in response to abiotic stress, and both exogenous application of betaine and the introduction via transgenes of the betaine-biosynthetic pathway into plants that do not naturally accumulate betaine increase the tolerance of these plants to various types of abiotic stress, such as drought, high salinity, and temperature stress [3].

In humans, betaine is obtained from the diet [2] or from its metabolic precursor choline [4]. Betaine is utilized as a methyl donor in a reaction that converts homocysteine into methionine via betaine-homocysteine methyltransferase. Betaine also plays a role in osmotic regulation in the kidneys, which are routinely exposed to high extracellular osmolarity during normal operation of the urinary concentrating mechanism [5]. Furthermore, dietary betaine suppresses the activation of nuclear factor-κB (NF-κB) with oxidative stress, and the protein expression of proinflammatory molecules such as cyclooxygenase-2 (COX-2), inducible nitric oxide synthase (iNOS), and tumor necrosis factor (TNF)-α in aged rat kidneys [6,7].

Betaine/GABA transporter-1 (BGT-1), the mouse transporter homologue of which is known as GABA transporter 2 (GAT2), is an integral membrane transporter capable of utilizing both betaine and GABA as substrates [8,9]. The distribution pattern of GAT2 mRNA does not closely match that of GABAergic pathways [8]. In a culture study, Olsen et al. [10] suggested that astroglial GAT2 expression and function are regulated by hyperosmolarity. Zhu & Ong [11] reported that BGT-1 expression is upregulated after kainite-induced neuronal injury in rat hippocampus. These reports suggested that GAT2/BGT-1 plays a role in osmoregulation in neural cells and that upregulation of GAT2/BGT-1 expression contributes to astrocytic swelling after brain injury. Interestingly, since GAT2 is co-localized with P-glycoprotein, a blood-brain barrier (BBB)-specific marker, in brain capillaries [12], it may also be involved in betaine transport across the BBB. These data suggest that betaine attenuates inflammatory processes and/or oxidative stress; however, the effects of betaine on central nervous system function in animals are poorly understood.

Lipopolysaccharide (LPS), a component of the cell wall of Gram-negative bacteria, is used to experimentally induce memory impairment, neuroinflammatory responses, and oxidative stress such as increases in mRNA levels of interleukin (IL)-1ß and IL-6 [13], heme oxygenase-1, microglial activation [14], and iNOS activity in hippocampus [15]. As neuroinflammation and oxidative stress are critical components of the pathogeneses of some neurodegenerative disorders, including Alzheimer's disease [16-18], and induce learning and memory impairment in rats [14], it is important to elucidate whether betaine improves LPS-induced memory impairment in order to understand the mechanism of action of betaine in the central nervous system.

In this study, we investigated the effects of betaine on LPS-induced memory impairment using the Y-maze and novel object recognition tests. We also examined the effect of betaine on LPS-induced changes in mRNA expression levels of proinflammatory molecules, glial markers, and GAT2 using real-time RT-PCR.

## Methods

### Animals

Male ddY strain mice (7-9 weeks old, 26 g - 44 g; Japan SLC., Hamamatsu, Japan) were used. The mice were kept in a regulated environment (24 ± 1°C, 55 ± 5% humidity) under a 12-h light/dark cycle (lights on 7:45 a.m.) and given food and tap water ad libitum. The experimental protocols concerning the use of laboratory animals were approved by the animal ethics board of Meijo University and followed the guidelines of the Japanese Pharmacological Society (Folia Pharmacol. Japon, 1992, 99: 35A); the Interministerial Decree of May 25th, 1987 (Ministry of Education, Japan); and the National Institutes of Health Guide for the Care and Use of Laboratory Animals (NIH Publications No. 8023, revised 1978). All efforts were made to minimize animal suffering and to reduce the number of animals used.

### Drugs

Betaine hydrochloride (betaine; Sigma, St. Louis, MO, USA) was dissolved in 0.9% saline and injected subcutaneously (s.c.). Lipopolysaccharide from *Escherichia coli *0111:B4 (LPS; Sigma) was dissolved in 0.9% saline and administered intracerebroventricularly (i.c.v.) into the lateral ventricle of the mouse brain according to the method of Haley & McCormick [19] at a dose of 5 μL/mouse under brief ether anesthesia. I.c.v. injections of LPS or saline were delivered at a rate of 5 μL/15 sec and injection needles were left in place an additional 10 sec. The total injection volume into the lateral ventricle was based on previous reports [13] and we confirmed that there are no influences of i.c.v. injection of saline (5 μL) itself on mouse behavior. The sham control animals were administered the vehicle (i.c.v. and s.c.) instead of one of the drug solutions.

### Experimental schedules

**Figure 1:**
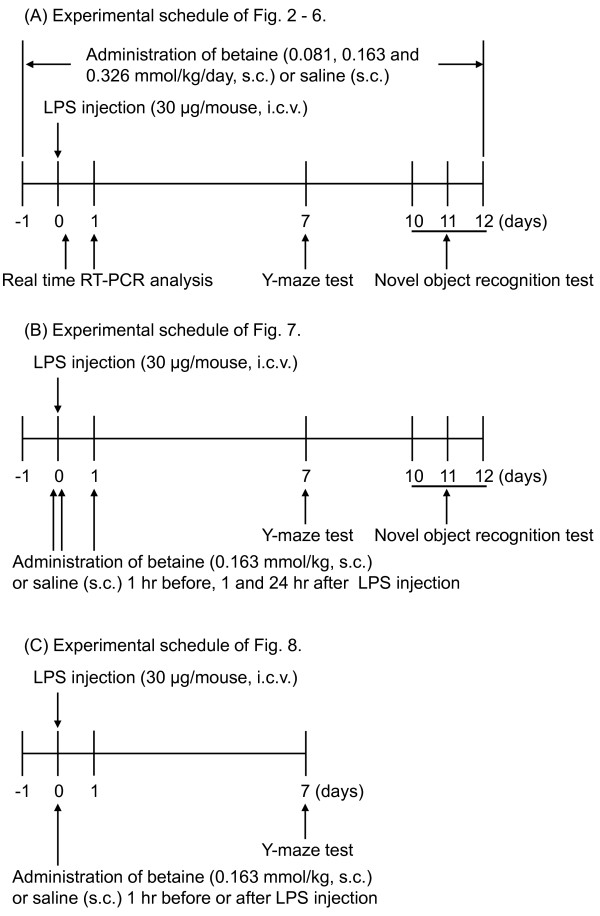
**Experimental schedules**.

First, we investigated whether betaine alleviated LPS-induced memory impairment using the Y-maze and novel object recognition tests, which were carried out 7 and 10-12 days after the LPS injection (30 μg/mouse, i.c.v.), respectively. Time schedules of behavioral experiments were referred to a previous report [15], which showed that LPS-induced memory impairment persists at least 15 days after LPS injection. To investigate the effects of repeated administration of betaine, mice were continuously treated with betaine (0.081, 0.163, or 0.326 mmol/kg, s.c.) for 13 days starting 1 day before LPS injection. On the day of the tests, betaine was administered 30 min before the start of the tests (Figure 1A). Proinflammatory molecules and glial activation are important for the pathogenesis of LPS-induced memory impairment, so we measured LPS-induced changes in mRNA expression of proinflammatory molecules and glial markers. The expression of each mRNA was measured 6 hr (proinflammatory molecules) or 24 hr (glial markers and betaine transporter) after LPS injection (Figure 1A). To investigate the effects of subacute administration of betaine, mice were treated with betaine (0.163 mmol/kg, s.c.) 1 hr before, 1 and 24 hr after LPS injection (Figure 1B).

### Spontaneous alternation performance (Y-maze test)

Immediate working memory was assessed by recording spontaneous alternation behavior during a single session in a Y-maze [20] made of black painted wood. Each arm was 40 cm long, 12 cm high, 3 cm wide at the bottom, 10 cm wide at the top, and converged in an equilateral triangular central area. The procedure was similar to that described previously [21]: each mouse, none of which had any prior experience with the maze, was placed at the end of one arm and allowed to move freely through the maze during an 8-min session, and arm entries were counted. Each series of arm entries was recorded visually, and an arm entry was defined as when the hind paws of the mouse were completely within the arm. Alternation was defined as successive entries into the three arms in overlapping triplet sets. The percentage alternation was calculated using the following formula:

$$\frac{number\;of\;alternations}{total\;number\;of\;arm\;entries-2}\times 100\%$$

### Novel object recognition test

The novel object recognition test, which was described previously [22], was used with some modifications. The apparatus consisted of a wooden open-field box (30 × 30 × 35 cm high). The task was divided into three different sessions (the habituation, familiarization, and retention sessions) and carried out for three consecutive days. On the first and second days, the mice were habituated to the experimental conditions and open-field apparatus without objects for 15 min/day. On the third day, the mice participated in a 5-min familiarization session in the presence of two identical objects (cylindrical columns). The time spent exploring each object, which was defined as when a mouse orientated their head toward the object and approached it (within 1 cm), was assessed manually using a stopwatch. Immediately after the familiarization session, the mice were removed from the apparatus, and one of the familiar objects was randomly replaced with a novel object (triangle pole). The mice were then returned to the apparatus and participated in a 5-min retention session in the presence of the familiar object and the novel object. The time spent exploring the familiar and novel objects was manually measured for 5 min. Then, an exploratory preference value was calculated; i.e., the ratio of the amount of time spent exploring any one of the two familiar objects (familiarization session) or the novel object (retention session) over the total time spent exploring the two types of objects. An exploratory preference of 50% corresponds to chance, and a significantly higher exploratory preference reflects good recognition memory.

### Real-time RT-PCR

For real-time RT-PCR, mice were sacrificed after the administration of LPS and/or betaine. Immediately after their decapitation, their hippocampi were rapidly dissected according to the method of Glowinski & Iversen [23] and immersed in liquid nitrogen. Frozen hippocampi were stored at -80°C until use. Total RNA was extracted using RNA-Bee Reagent (Tel-Test, Inc., Friendswood, TX, USA) according to the manufacturer's instructions, which is an improved version of the single-step method of RNA isolation [24]. Reverse transcription was performed with an ExScript RT reagent Kit (Perfect Real Time) or a PrimeScript RT reagent Kit (Perfect Real Time) (Takara Bio Inc., Otsu, Japan) under the conditions recommended by the manufacturer. Real-time PCR analysis was undertaken using SYBR Premix Ex Taq or SYBR Premix Ex Taq II (Takara Bio Inc.). Data collection involved using a Chromo4 real-time PCR detector and analysis with an Opticon Monitor 3 (Bio-Rad laboratories Inc., Hercules, CA, USA). The real-time PCR primers used in this study are listed in Table 1. All primers were purchased from Takara Bio Inc. The real-time PCR conditions were as follows: initial denaturation at 95°C for 10 s followed by 40 cycles of 95°C for 5 s and 60°C for 20 s. The expression levels of the genes analyzed by real-time PCR were quantified by comparison with a standard curve and normalized relative to levels of ß-actin.

**Table 1:** Gene-specific real time RT-PCR primer sequences.

Gene		Sequence (5'-3')
ß-actin	forward	TGACAGGATGCAGAAGGAGA
	reverse	GCTGGAAGGTGGACAGTGAG
CD11b	forward	TCACCCTCAAGGGCAACCTATC
	reverse	AGGGCAAACGCAGAGTCATTAAAC
CD45	forward	TCCCAGCAGACAGGGTTGTTC
	reverse	GTCCATTCTGGGCGGGATAG
COX-2	forward	GTGTGCGACATACTCAAGCAGGA
	reverse	TGAAGTGGTAACCGCTCAGGTG
GAT2	forward	CCATCTTGGGCTTCATGTCTCA
	reverse	CAGCTGGGACAAAGGCATCA
GFAP	forward	ACCAGCTTACGGCCAACAGTG
	reverse	TGTCTATACGCAGCCAGGTTGTTC
IL-1ß	forward	TCCAGGATGAGGACATGAGCAC
	reverse	GAACGTCACACACCAGCAGGTTA
IL-6	forward	CCACTTCACAAGTCGGAGGCTTA
	reverse	GCAAGTGCATCATCGTTGTTCATAC
iNOS	forward	GGAATGGAGACTGTCCCAGCA
	reverse	GTCATGAGCAAAGGCGCAGA
Heme oxygenase-1	forward	TGCAGGTGATGCTGACAGAGG
	reverse	TGTCTGGGATGAGCTAGTGCTGA
TNF-α	forward	AAGCCTGTAGCCCACGTCGTA
	reverse	GGCACCACTAGTTGGTTGTCTTTG

### Data analysis

Statistical analysis was performed, and the figures were produced using Prism 5 for Mac OS X (GraphPad Software, Inc., San Diego, CA, USA). It could not be assumed that the behavioral data were sampled from a Gaussian distribution; therefore, the data are expressed as median and interquartile range values. Significance was evaluated using the Mann-Whitney U-test for comparisons between two groups, and Kruskal-Wallis non-parametric one-way ANOVA followed by Bonferroni's test were used for multiple comparisons. The expression levels of each mRNA are shown as mean ± S.E.M. An unpaired t-test (also with Welch-correction when F-test was significant) was used to compare two groups, and one-way ANOVA followed by Dunnett's test was used for multiple comparisons. The criterion for significance was p < 0.05.

## Results

### Effects of repeated administration of betaine on LPS-induced memory impairment

**Figure 2:**
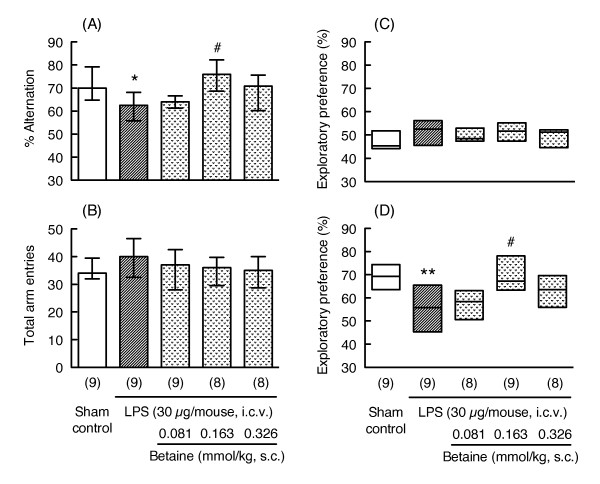
**Effects of repeated administration of betaine on LPS-induced memory impairment**. Y-maze and novel object recognition tests were carried out 7 and 10-12 days after LPS injection (30 μg/mouse, i.c.v.), respectively. The mice were continuously treated with betaine (0.081, 0.163 and 0.326 mmol/kg, s.c.) for 13 days starting 1 day before LPS injection. On the day of the Y-maze and novel object recognition tests, betaine was administered 30 min before the test. Y-maze data (A: % alternation, B: total arm entries) are shown as the median (vertical column) and as the first and third quartile values (vertical line). Novel object recognition data (C: familiarization session, D: retention session) are shown as the median (horizontal bar) and as the first and third quartile values (vertical column). The number of mice used is shown in parentheses. Significance levels: *p < 0.05, **p < 0.01 vs. sham control (Mann-Whitney's U-test), and #p < 0.05 vs. LPS alone (Bonferroni's test).

In the Y-maze test, LPS treatment (30 μg/mouse, i.c.v.) significantly decreased the percentage of alternations 7 days after LPS injection (Mann-Whitney U-test, p < 0.05, U = 17.00, Figure 2A) without changing the total number of arm entries (Mann-Whitney U-test, p = 0.199, U = 25.50, Figure 2B). Repeated administration of betaine showed a bell-shaped dose-response relationship, and a dose of 0.163 mmol/kg (s.c.) significantly reversed the LPS-induced impairment of spontaneous alternations (Bonferroni's test, p < 0.05, Figure 2A) without changing the total number of arm entries (Kruskal-Wallis non-parametric ANOVA, H(3) = 2.021, p = 0.568, Figure 2B).

In the novel object recognition test, there was a decrease in preference for the novel object (Mann-Whitney U-test, p < 0.01, U = 11.00, Figure 2D) without any changes in exploratory behavior during the familiarization session (Exploratory preference: Mann-Whitney U-test, p = 0.222, U = 26.00, Figure 2C; Total exploratory time: Mann-Whitney U-test, p = 0.610, U = 34.00, Table 2) 12 days after injection of LPS (30 μg/mouse). Repeated administration of betaine also showed a bell-shaped dose-response relationship, as was shown in the Y-maze test, and the same dose of betaine (0.163 mmol/kg) significantly reversed the LPS-induced decrease in exploratory behavior (Bonferroni's test, p < 0.05, Figure 2D) without any changes in exploratory behavior during the familiarization session (Exploratory preference: Kruskal-Wallis non-parametric ANOVA, H(3) = 2.033, p = 0.566, Figure 2C; Total exploratory time: Kruskal-Wallis non-parametric ANOVA, H(3) = 0.4513, p = 0.929, Table 2).

**Table 2:** Total exploratory time in the familiar session.

Treatment	N	Total exploratory time (sec) (range)
Sham control	9	10.73 (7.865 - 11.66)
LPS (30 μg/mouse, i.c.v.)	9	9.980 (6.980 - 12.12)
LPS (30 μg/mouse, i.c.v.) + betaine (0.081 mmol/kg, s.c.)	8	10.94 (7.128 - 12.43)
LPS (30 μg/mouse, i.c.v.) + betaine (0.163 mmol/kg, s.c.)	9	10.24 (6.295 - 11.99)
LPS (30 μg/mouse, i.c.v.) + betaine (0.326 mmol/kg, s.c.)	8	9.325 (7.215 - 10.75)

See Fig. 2 for details.

### Effects of betaine on LPS-induced increases in mRNA expression of proinflammatory molecules

**Figure 3:**
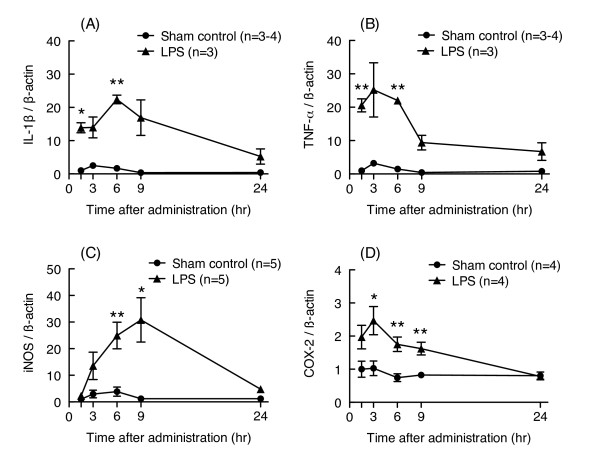
**LPS-induced changes in levels of mRNA related to inflammation in the hippocampus**. Time-dependent changes in mRNA expression levels for IL-1β, TNF-α, iNOS, and COX-2 in hippocampus after LPS injection are shown in figures (A), (B), (C), and (D), respectively. LPS (30 μg/mouse, i.c.v.) or saline was injected into the lateral ventricle of each mouse. The mice were sacrificed 1.5, 3, 6, 9, or 24 hr after LPS injection. Their mRNA levels were assessed by real-time RT-PCR. Each mRNA level was normalized to the mRNA level of β-actin as an endogenous control. Values are shown as the mean ± S.E.M. for 3-5 mice, as shown in parentheses. Significance levels: *p < 0.05, **p < 0.01 vs. corresponding sham control (unpaired t-test).

**Figure 4:**
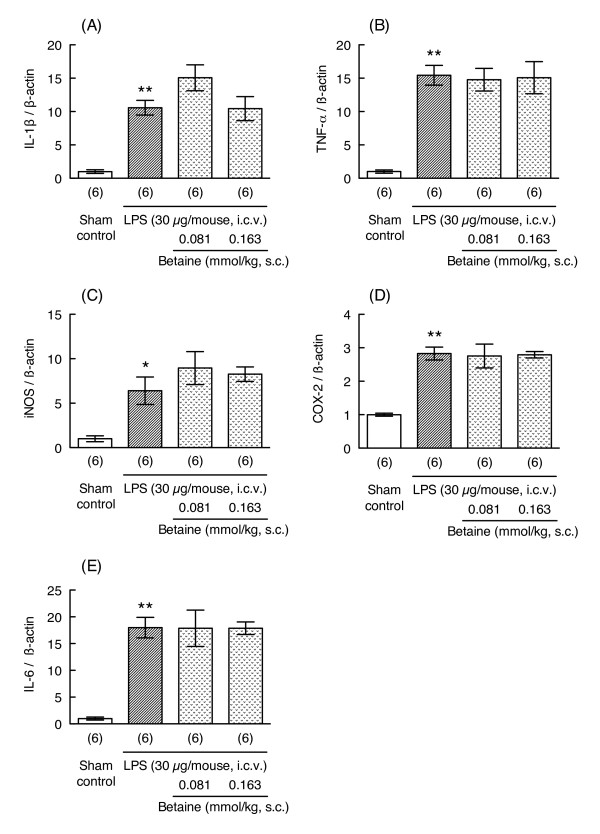
**Effects of betaine on LPS-induced increases in the levels of mRNA related to inflammation**. Mice were treated with betaine (0.081 and 0.163 mmol/kg, s.c.) 24 hr before and immediately before LPS injection (30 μg/mouse, i.c.v.), and sacrificed 6 hr after LPS injection. mRNA levels in hippocampus were assessed by real-time RT-PCR. The level of each mRNA was normalized to the mRNA level of β-actin as an endogenous control. Values are shown as the mean ± S.E.M. for 6 mice, as shown in parentheses. Significance levels: *p < 0.05, **p < 0.01 vs. sham control (unpaired t-test).

Cytokines and proinflammatory molecules are important for the pathogenesis of LPS-induced memory impairment. We therefore investigated whether repeated administration of betaine could prevent LPS-induced increases in mRNA expression levels for proinflammatory molecules such as IL-1β, TNF-α, iNOS, and COX-2. The mRNA expression levels of these inflammatory molecules transiently increased after LPS injection and recovered to baseline levels by 24 hr after LPS injection (Figure 3). LPS treatment (30 μg/mouse) significantly increased the mRNA expression levels of IL-1β, TNF- α, iNOS, COX-2, and IL-6 6 hr after LPS injection (unpaired t-test, p < 0.05 vs. corresponding sham control group, t = 8.451, 9.591, 3.413, 9.164 and 8.749, respectively, df = 5, Figure 4). Administration of betaine (0.081 and 0.163 mmol/kg) did not prevent the LPS-induced increases in the levels of these mRNAs (one-way ANOVA; IL-1β: F_2, 15 _= 2.535, p = 0.113; TNF- α: F_2, 15 _= 0.0308, p = 0.970; iNOS: F_2, 15 _= 0.8014, p = 0.467; COX-2: F_2, 15 _= 0.0228, p = 0.978; IL-6: F_2, 15 _= 0.0009, p = 0.999; Figure 4). The mRNA expression level of heme oxygenase-1, a known marker of oxidative stress, was also significantly increased 6 hr after LPS injection (unpaired t-test, p < 0.05; Sham control group: 1.000 ± 0.084, n = 4; LPS group: 3.688 ± 0.520, n = 4, Welch-corrected t = 5.101, df = 3), and betaine treatment (0.163 mmol/kg) did not prevent this increase (unpaired t-test, p = 0.961, t = 0.0508, df = 7; LPS group: 3.688 ± 0.520, n = 4; LPS + betaine group: 3.730 ± 0.608, n = 5).

### Effects of betaine on LPS-induced increases in mRNA expression levels of glial markers and the betaine transporter

**Figure 5:**
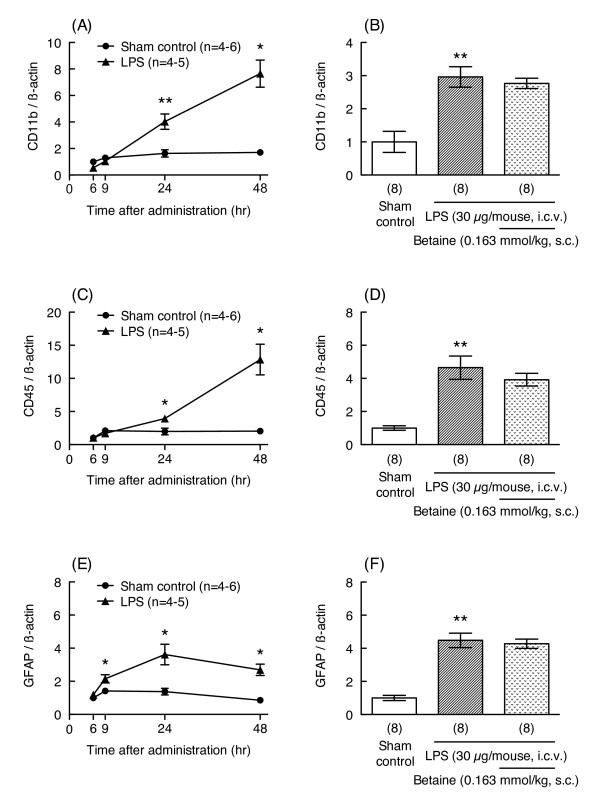
**Effects of betaine on LPS-induced increases in mRNA expression levels for glial markers**. Time-dependent changes in mRNA expression levels for CD11b, CD45, and GFAP in hippocampus 6, 9, 24, and 48 hr after LPS injection (30 μg/mouse, i.c.v.) are shown in figures (A), (C), and (E), respectively. The mice were treated with betaine (0.163 mmol/kg, s.c.) 24 hr before and immediately before LPS injection (30 μg/mouse, i.c.v.), and sacrificed 24 hr after LPS injection. mRNA levels in hippocampus were assessed by real-time RT-PCR. The level of each mRNA was normalized to the mRNA level of β-actin as an endogenous control. Values are shown as the mean ± S.E.M. for 4-8 mice, as shown in parentheses. Significance levels: *p < 0.05, **p < 0.01 vs. corresponding sham control (unpaired t-test).

Glial activation is also involved in the pathogenesis of LPS-induced memory impairment; therefore, to understand the effects of betaine on these cells, LPS-induced increases in mRNA expression levels for CD11b and CD45, which are microglial markers, and glial fibrillary acidic protein (GFAP), a marker of astrocytes, were investigated. LPS treatment (30 μg/mouse) significantly increased mRNA expression levels of CD11b, CD45, and GFAP 24 hr after injection (unpaired t-test, p < 0.01, t = 4.425, df = 14 for CD11b; Welch-corrected t = 5.083, df = 7 for CD45; Welch-corrected t = 7.528, df = 8 for GFAP, Figure 5); however, betaine treatment (0.163 mmol/kg) did not prevent LPS-induced increases in mRNA levels of these glial markers (unpaired t-test, p = 0.5603, df = 14 for CD11b; p = 0.9085, df = 14 for CD45; t = 0.3956, df = 14 for GFAP, Figure 5).

**Figure 6:**
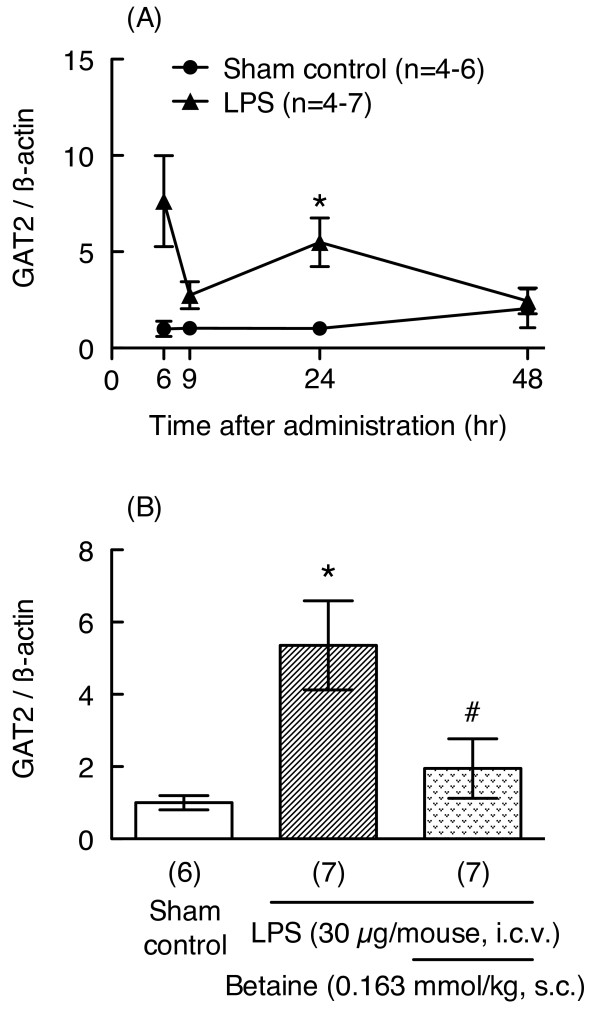
**Effects of betaine on LPS-induced increases in mRNA expression levels for GAT2**. Time-dependent changes in GAT2 mRNA expression in hippocampus 6, 9, 24, and 48 hr after LPS injection (30 μg/mouse, i.c.v.) are shown in the upper panel (A). Mice were treated with betaine (0.163 mmol/kg, s.c.) 24 hr before and immediately before LPS injection (30 μg/mouse, i.c.v.), and sacrificed 24 hr after LPS injection. mRNA levels in hippocampus were assessed by real-time RT-PCR. The level of each mRNA was normalized to the mRNA level of β-actin as an endogenous control. Values are shown as the mean ± S.E.M. for 4-7 mice, as shown in parentheses. Significance levels: *p < 0.05 vs. sham control, #p < 0.05 vs. LPS alone (unpaired t-test).

Betaine may act on GAT2/BGT-1 expressed in neurons and/or glial cells to improve memory impairment; therefore, we examined the effects of LPS and betaine on mRNA expression for GAT2. LPS treatment (30 μg/mouse) significantly increased mRNA expression for GAT2 24 hr after injection (unpaired t-test, p < 0.05, Welch-corrected t = 3.489, df = 6, Figure 6A, B). Interestingly, betaine (0.163 mmol/kg) prevented this LPS-induced increase in GAT2 mRNA levels (unpaired t-test, p < 0.05, t = 2.301, df = 12, Figure 6B). These results may indicate that repeated administration of betaine is not necessary to prevent LPS-induced memory impairment. Therefore, as our next experiment, we conducted behavioral experiments after subacute (1 hr before, 1 and 24 hr after LPS injection) or acute (1 hr before or after LPS injection) administration of betaine.

### Effects of subacute administration of betaine on LPS-induced memory impairment

**Figure 7:**
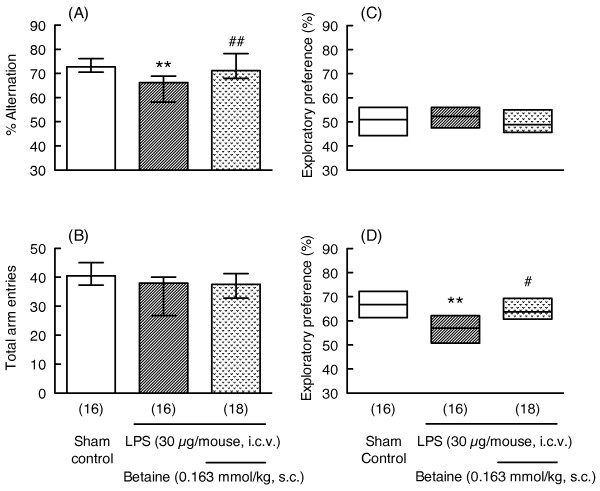
**Effects of subacute administration of betaine on LPS-induced memory impairment**. Y-maze and novel object recognition tests were carried out 7 and 10-12 days after LPS injection (30 μg/mouse, i.c.v.), respectively. Mice were treated with betaine (0.163 mmol/kg, s.c.) 1 hr before and 1 and 24 hr after LPS injection. Y-maze data (A: % alternation, B: total arm entries) are shown as the median (vertical column) and the first and third quartile values (vertical line). The novel object recognition data (C: familiarization session, D: retention session) are shown as the median (horizontal bar) and the first and third quartile values (vertical column). The number of mice used is shown in parentheses. Significance levels: **p < 0.01 vs. sham control, #p < 0.05, ##p < 0.01 vs. LPS alone (Mann-Whitney's U-test).

LPS treatment (30 μg/mouse) significantly decreased the percentage of alternations in the Y-maze test (Mann-Whitney U-test, p < 0.01, U = 59.0, Figure 7A) and the degree of preference for the novel object (Mann-Whitney U-test, p < 0.01, U = 58.0, Figure 7D). Subacute administration of betaine (0.163 mmol/kg) significantly reversed LPS-induced memory impairment in the Y-maze (Mann-Whitney U-test, p < 0.01, U = 64.0, Figure 7A) and novel object recognition tests (Mann-Whitney U-test, p < 0.05, U = 70.0, Figure 7D). These treatments had no influences on the total number of arm entries in the Y-maze test (Figure 7B) or on exploratory behavior during the familiarization session in the novel object recognition test (Figure 7C, Table 3).

**Table 3:** Total exploratory time in the familiar session.

Treatment	N	Total exploratory time (sec) (range)
Sham control	16	8.600 (5.918 - 10.40)
LPS (30 μg/mouse, i.c.v.)	16	7.810 (6.813 - 9.165)
LPS (30 μg/mouse, i.c.v.) + betaine (0.163 mmol/kg, s.c.)	18	8.055 (5.540 - 10.87)

See Fig. 7 for details.

### Effects of acute administration of betaine on LPS-induced memory impairment

**Figure 8:**
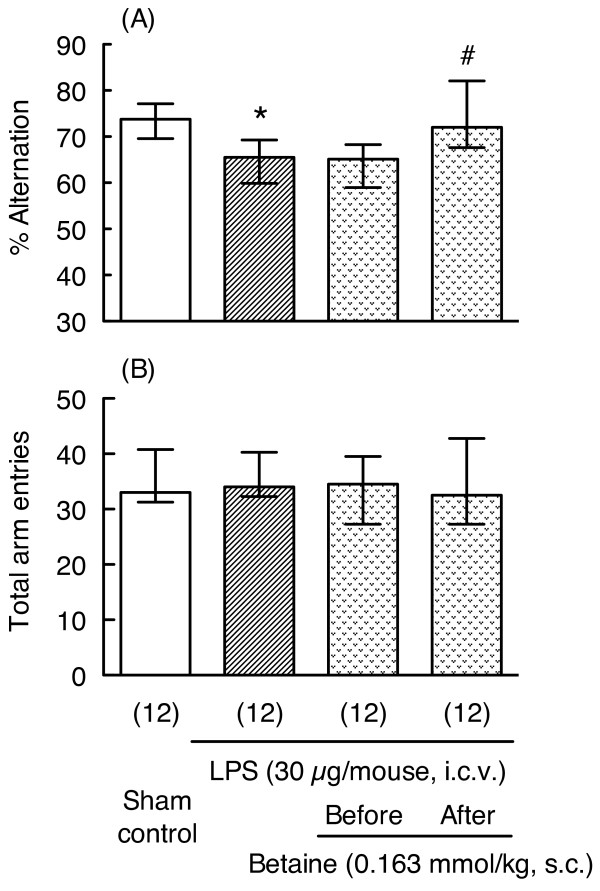
**Effects of acute administration of betaine on LPS-induced memory impairment in the Y-maze test**. The Y-maze test was carried out 7 days after LPS (30 μg/mouse, i.c.v.) injection. The mice were treated with betaine (0.163 mmol/kg, s.c.) 1 hr before or 1 hr after LPS injection. Y-maze data (A: % alternation, B: total arm entries) are shown as the median (vertical column) and the first and third quartile values (vertical line). The number of mice used is shown in parentheses. Significance levels: *p < 0.05 vs. sham control (Mann-Whitney's U-test), #p < 0.05 vs. LPS alone (Mann-Whitney's U-test).

We further examined whether a single administration of betaine is able to prevent LPS-induced memory impairment (experimental schedule shown in Figure 1C). Interestingly, a single administration of betaine (0.163 mmol/kg) 1 hr after LPS injection also significantly reversed LPS-induced impairment of spontaneous alternation (Mann-Whitney U-test, p < 0.05, U = 29.5, Figure 8A); however, a single administration of betaine 1 hr before LPS injection did not reverse LPS-induced impairment of spontaneous alternation (Mann-Whitney U-test, p = 0.795, U = 67.0, Figure 8A).

## Discussion

It has been reported that betaine suppresses expression of proinflammatory molecules such as COX-2, iNOS, and TNF- α; and increases oxidative stress in aged rat kidney [6,7]. Betaine also prevents chronic ethanol consumption-induced oxidative stress in brain synaptosomes [25]. These reports suggest that betaine might be a useful compound for preventing neurodegenerative disorders and/or other diseases involving inflammatory processes and oxidative stress; however, the effects of betaine on memory impairment involving neuroinflammatory and/or oxidative stress are not well known. Therefore, the effects of betaine on LPS-induced memory impairment were evaluated. Repeated administration of betaine (0.163 mmol/kg) improved LPS-induced memory impairment in the Y-maze and novel object recognition tests, with a bell-shaped dose-response relationship. Our findings suggest that betaine improves LPS-induced memory impairment, but it is possible that the preference for the object changed due to some perceptual effects rather than memory effects, and/or induction of sickness behavior may have changed the innate preference for an object without affecting memory processes. However, we used identical objects in the familiarization sessions, after which one of these objects was randomly replaced with a novel object. Further, sickness behavior is usually assessed within 24 hr of induction, but in our protocol the behavioral experiments were conducted 7 to 12 days after LPS injection. On these days, no sickness-like behavior was seen, as in other investigations; therefore, we think that the effects of LPS and/or betaine reflect memory function rather than other effects. Taken together, these results suggest that betaine has a preventative effect on LPS-induced memory impairment caused by neuroinflammatory responses.

As described in Background, LPS induces expression of proinflammatory molecules and glial activation within several days of LPS injection. For example, Szczepanik & Ringheim [26] reported that i.c.v. injection of LPS induces production of proinflammatory cytokines such as IL-1α, IL-1β, IL-6, and TNF-α in mouse hippocampus and cortex. These increases in the expression levels of proinflammatory cytokines peaked about 6 - 9 hr after LPS injection. LPS-induced neuronal injury requires the presence of microglia and Toll-like receptor 4-dependent pathways [27]. Choi et al. [28] reported that i.c.v. injection of LPS induces neuronal damage and activation of microglia and astrocytes in hippocampus 24 hr after LPS injection. Therefore, we investigated whether betaine could suppress LPS-induced increases in mRNA expression levels of various proinflammatory molecules and glial markers in hippocampus concurrently with the observed improvements in memory impairment. LPS induced a transient increase in mRNA expression levels for IL-1β, TNF- α, iNOS, and COX-2; and these increases returned to sham-control levels by 24 hr after LPS injection; however, betaine (0.081 or 0.163 mmol/kg) did not affect the LPS-induced increases in mRNA levels for these inflammatory molecules.

LPS treatment (30 μg/mouse) also increased mRNA expression levels of the microglial markers CD11b and CD45, and the astrocytic marker GFAP; however, betaine also did not prevent the LPS-induced increases in mRNA levels for these glial markers. Our results indicate that betaine does not suppress mRNA expression of proinflammatory molecules or glial markers, and the mechanism behind the ameliorating effects of betaine on memory impairment is not mediated by the expression of these genes, which is the mechanism by which betaine suppresses the expression of proinflammatory molecules and increased oxidative stress in aged rat kidney [6,7]. This finding indicates that the mechanism behind the actions of betaine in the central nervous system is different from that in kidney.

Four different subtypes of GAT have been cloned and are termed GAT1, GAT2, GAT3, and GAT4 in mice (GAT-1, BGT-1, GAT-2 and GAT-3, respectively, in rats and humans) [29]. GAT2/BGT-1 transports both GABA and betaine [9,30]. In renal epithelial cells, GAT2/BGT-1 is a basolateral membrane protein that protects cells in the hypertonic inner medulla by mediating betaine uptake and accumulation [5]. In the central nervous system, it has been reported that betaine content and BGT-1 mRNA levels are increased in brain of rats with hyperosmotic serum induced by the injection and drinking of NaCl solution [31,32]. In addition, protein and mRNA expressions of GAT2/BGT-1 are upregulated in mouse and rat astrocyte primary cultures exposed to hyperosmotic conditions [10,33]. These results suggest that betaine and GAT2/BGT-1 play important roles in osmotic regulation in the central nervous system. Moreover, expression of BGT-1 is increased in astrocytes after kainate-induced neuronal injury in rat hippocampus [11]. While betaine and GAT2/BGT-1 may be involved in neuronal dysfunction caused by neurodegeneration or neuronal injury, their physiological roles are not yet known. In the present study, we examined mRNA expression for GAT2 after treatment with LPS and/or betaine in mouse hippocampus. LPS treatment (30 μg/mouse) significantly increased mRNA expression for GAT2 24 hr after LPS injection. Interestingly, betaine (0.163 mmol/kg) blocked this LPS-induced increase in mRNA expression for GAT2, suggesting that betaine and its transporter, GAT2/BGT-1, play important roles in neuronal dysfunction caused by neuronal injury.

It is known that the changes that occur during the early phase after LPS treatment are crucial to delayed neuronal impairment such as the memory impairment shown in this study. To elucidate the mechanisms underlying the effects of betaine, we considered that administration of betaine during the early phase after LPS injection might be necessary for preventing LPS-induced memory because mRNA expression levels for GAT2 transiently increased after LPS injection and recovered by 48 hr after LPS injection. Interestingly, either subacute (1 hr before, 1 and 24 hr after the LPS injection) or single (1 hr after the LPS injection) administration of betaine prevented LPS-induced memory impairment, but this effect was not seen when betaine was given 1 hr before LPS injection. Consistent with betaine's effect in alleviating LPS-induced delayed memory impairment, betaine also significantly reduced LPS-induced increases in GAT2 mRNA levels in hippocampus. These data suggest that during the early period after LPS injection, betaine plays a crucial role in preventing LPS-induced neuronal dysfunction. On the other hand, a single administration of betaine, 1 hr before LPS injection, did not prevent LPS-induced memory impairment. This finding that betaine has a neuroprotective effect on delayed memory impairment even when administered after LPS injection has important therapeutic implications. Excitotoxicity has been implicated in the etiology of ischemic stroke and chronic neurodegenerative disorders. Hence, the development of novel neuroprotective molecules that ameliorate excitotoxic brain damage is being vigorously pursued. Indeed, betaine attenuates glutamate-induced neurotoxicity in primary cultured brain cells [34]. Montoliu et al. [35] reported that a family of trialkylglycines significantly prevent excitotoxic neuronal death in models of neurodegeneration. Since dietary and supplementary administration of betaine has been studied in humans, if the detailed mechanism of betaine could be clarified, it could become a candidate for treatment of cognitive dysfunction in disorders such as Alzheimer's disease and senile dementia.

## Conclusions

Betaine improves LPS-induced memory impairment and blocks LPS-induced increases in mRNA expression for GAT2; however, betaine does not prevent LPS-induced increases in mRNA expression of proinflammatory molecules or glial markers. These results suggest that betaine has protective effects against LPS-induced memory impairment that are mediated through unique mechanisms involving betaine actions on GAT2, which is involved in the development of memory impairment, without affecting proinflammatory molecules or glial markers.

## Competing interests

The authors declare that they have no competing interests.

## Authors' contributions

MT carried out the behavioral experiments. YN and AE carried out the real-time RT-PCR. MM participated in the design of the study, performed the statistical analysis, drafted the manuscript, and helped to carry out the behavioral experiments and real-time RT-PCR. MH conceived the study, participated in its design and coordination, and helped to draft the manuscript. All of the authors have read and approved the final manuscript.
